# Supplementary material for: Time-resolved NMR monitoring of tRNA maturation
Source: Nat Commun. 2019 Jul 29;10:3373. doi: 10.1038/s41467-019-11356-w (PMC6662845; doi:10.1038/s41467-019-11356-w)
Supplement: Supplementary file 1 — Supplementary Information [file 41467_2019_11356_MOESM1_ESM.pdf]

Time-resolved NMR monitoring of tRNA maturation

Content:

Supplementary Tables 1 to 4 pages 2-5
Supplementary Figures 1 to 11 pages 6-16
Supplementary References page 17

Supplementary Table 1: Overview of the 14 modifications present in yeast cytoplasmic $tRNA^{Phe}$

modification	enzyme	gene	partner protein	localization
**m^2G10**	Trm11	*trm11*	Trm112	cytoplasm
**D16**	Dus1	*dus1*	-	nucleus
**D17**	Dus1	*dus1*	-	nucleus
**m^2_2G26**	Trm1	*trm1*	-	nucleus
**Cm32**	Trm7	*trm7*	Trm732	cytoplasm
**Gm34**	Trm7	*trm7*	Trm734	cytoplasm
**yW37**	Trm5, TYW1-4	*trm5, tyw1-4*	-	nucleus/cytoplasm[a]
**Ψ39**	Pus3	*pus3*	-	nucleus
**m^5C40**	Trm4	*trm4*	-	nucleus
**m^7G46**	Trm8	*trm8*	Trm82	nucleus
**m^5C49**	Trm4	*trm4*	-	nucleus
**m^5U54 (T54)**	Trm2	*trm2*	-	nucleus[b]
**Ψ55**	Pus4	*pus4*	-	nucleus
**m^1A58**	Trm61	*trm61*	Trm6	nucleus

Modifications are listed in the first column, the catalytic enzyme responsible for their introduction in the second column and the corresponding genes in the third column. Partner proteins, when present are listed in the fourth column. The known sub-cellular localizations (nucleus or cytoplasm) of the enzymes are given in the fifth column.

[a] The complex modification yW37 (wybutosine37) requires five enzymes for its biosynthesis, *i.e.* Trm5 and TYW1-4, which are found in the nucleus for Trm5 and cytoplasm for TYW1-4 (1).

[b] from indirect evidence (2,3).

Supplementary Table 2: Strains used in this study

strain	genotype	source
BY4741	*MATa his3-Δ1 leu2-Δ0 met15-Δ0 ura3-Δ0* (wild type)	Euroscarf
BY4741-*dus1Δ*	BY4741, *YML080w::kanMX*	Euroscarf
BY4741-*pus4Δ*	BY4741, *YNL292w::kanMX*	Euroscarf
BY4741-*trm1Δ*	BY4741, *YDR120c::kanMX*	Euroscarf
BY4741-*trm2Δ*	BY4741, *YKR056w::kanMX*	Euroscarf
BY4741-*trm4Δ*	BY4741, *YBL024w::kanMX*	Euroscarf
BY4741-*trm8Δ*	BY4741, *YDL201w::kanMX*	Euroscarf
BY4741-*trm11Δ*	BY4741, *YOL124c::kanMX*	Euroscarf
c13-ABYS-86	*MATα ura3Δ5 leu2-3,112 his3 pra1-1 prb1-1 prc1-1 cps1-3* (wild-type)	(4)
c13-ABYS-86-*dus1Δ*	c13-ABYS-86, *YML080w::kanMX*	This study
c13-ABYS-86-*pus4Δ*	c13-ABYS-86, *YNL292w::kanMX*	This study
c13-ABYS-86-*trm1Δ*	c13-ABYS-86, *YDR120c::kanMX*	This study
c13-ABYS-86-*trm2Δ*	c13-ABYS-86, *YKR056w::kanMX*	This study
c13-ABYS-86-*trm4Δ*	c13-ABYS-86, *YBL024w::kanMX*	This study
c13-ABYS-86-*trm8Δ*	c13-ABYS-86, *YDL201w::kanMX*	This study
c13-ABYS-86-*trm11Δ*	c13-ABYS-86, *YOL124c::kanMX*	This study

Supplementary Table 3: Oligonucleotides used in this study

primer name	sequence
*dus1*_A	GCAAGGTGATCGTCAAACTGCACT
*dus1*_B	TATGCCTTGAGGACAGCCCAAA
*dus1*_C	TATTTCCAGATTGTCAAAGAATGCCAG
*dus1*_D	ATGGAGACGGAGTTGAACATTTTCT
*dus1*_UP	TTACAGGCTCGCAATAAGCTCAATAAACAATATCAACTGCATATGGATGTCCACGAGCTCTCT
*dus1*_DN	GAATAGAAAAGGCAAAGAAATATACATGGATTTTATCAGGTTTTAATCGATGAATTCGAGCTCG
*pus4*_A	TGGAATAATAGCATCAAGGAGAAAG
*pus4*_B	ACATCTCCTGAAGTAGTGGAAACAC
*pus4*_C	ACGTGAAGGAATGTATAGTTTCGAG
*pus4*_D	TTTCCTGTCCGAATGATCTTACTAC
*pus4*_UP	AGTTAGGGCATCACATAAAAAGAAAACTCAATCAAGGGGAACATG
*pus4*_DN	TTCACAAGACCAAAAAAAAAAATGGTTCGAACTTCCTCTTTTTTA
*trm1*_A	TAAATAAGCAATGAAAGGCTTGACT
*trm1*_B	ACTTATTATTCGTCGCTTTGTTACG
*trm1*_C	ACCTCTACACAACAAGGAGTTCATC
*trm1*_D	ATTTATATGCCCAGAGGTTACAACA
*trm1*_UP	TGAGCAGTCATAAGTTGATACCTTTCCTCTTACAATGTAGATATG
*trm1*_DN	CCTTAGTTTCTTACGTTTTAGCTCTAACACTAATCAAAATTCTTA
*trm2*_A	ATCAAGACGAAACAATTAGAACCTG
*trm2*_B	CGGTATGATAACAACTTGCTTCTTT
*trm2*_C	AAGTGAAAACACTTCCGTTATCTTG
*trm2*_D	TTTTCCATTTTGGTATGATGTTTCT
*trm2*_UP	CTGTCATTTTATTTTAGAGGAATAGTTTAGGACAAAGTCATTATG
*trm2*_DN	CACTCTCAACGTGGTGCGTTTGTGGAAAGAAATCAAATCCTCTTA
*trm4*_A	TCTTTTGGAAGAGATTAATTTGCAC
*trm4*_B	GGCTTCTTGTCTTGAGATATTACCA
*trm4*_C	TCAAAGAAGTGATATCGAATGTTCA
*trm4*_D	TGGGTCACTAAAACTTTGTTATTCC
*trm4*_UP90	CACCTACTATAGTTAAAAACATATTACTTATACTAGTTCTAACACTTCC
*trm4*_DN90	TCATTACTGAAGTATTATATTCTTATTTTGCCTTTTAATAATATACAT
*trm8*_A	CCTAAAGAGGATGTGGAGTCAACTA
*trm8*_B	ACTGGGTACTCTAACTGGTGATCTG
*trm8*_C	CAGATCACCAGTTAGAGTACCCAGT
*trm8*_D	TGATATTGCAAAGAGAATAGGGAAG
*trm8*_UP	GCAGTTCCCATAGGATAAAATTTTCAAGCGTTTATTGTTAAGATG
*trm8*_DN	AGTTATGTATATGTGGTAAATTGTTCTAGTTATACATCTATGTTA
*trm11*_A	TTAGAGGGTTTTTGATCTTCTTCCT
*trm11*_B	TTCATATCAATCTTTCCTTCAAAGC
*trm11*_C	AATGCACGAAAATCTAGAGCTAAAA
*trm11*_D	TAAGGTACGTAGAATCCGATGTTTC
*trm11*_UP	TAGCATAACTAAACACATTGTATATAAGGTAAAAACGGCAGCATG
*trm11*_DN	AAGTAAATATATCTTAAAATTACAAAGGGCTTATGCATCATTTCA
kanB	CTGCAGCGAGGAGCCGTAAT
kanC	TGATTTTGATGACGAGCGTAAT
$tRNA^{Phe}$_template	TGGTGCGAATTCTGTGGATCGAACACAGGACCTCCAGATCTTCAGTCTGGCGCTCTCCCAACTG AGCTAAATCCGCTATAGTGAGTCGTATTA
T7 promotor	TAATACGACTCACTATAG

Supplementary Table 4: Mass spectrometric parameters for detection of RNA nucleosides by isotope dilution mass spectrometry

Compound Group	Compound Name	Precursor Ion (m/z)	Product Ion (m/z)	Ret Time (min)	Delta Ret Time (min)	Fragmentor (V)	Collision Energy (eV)	Cell Accelerator Voltage (V)	Polarity
unlabelled	C	244.1	112.1	2.1	1	175	13	5	Positive
	U	245.1	113.1	2.9	1	95	5	5	Positive
	G	284.1	152.0	4.1	1	150	17	5	Positive
	A	268.1	136.1	5.2	1	200	25	5	Positive
	D	247.1	115.0	1.7	1	70	5	5	Positive
	Ψ	245.1	209.0	1.7	1	90	5	5	Positive
	m^1A	282.1	150.0	3.3	2	150	25	5	Positive
	m^5C	258.1	126.1	3.5	1	185	13	5	Positive
	Cm	258.0	112.0	3.8	1	180	9	5	Positive
	m^7G	298.1	166.0	3.8	1	100	13	5	Positive
	I	269.1	137.0	4.0	1	100	10	5	Positive
	m^5U	259.1	127.1	4.1	1	95	9	5	Positive
	Gm	298.1	152.0	4.9	1	100	9	5	Positive
	m^2G	298.1	166.0	5.0	1	95	17	5	Positive
	m^2_2G	312.1	180.0	5.6	1	130	13	5	Positive
	m^1G	298.1	166.0	4.8	1	130	13	5	Positive
	m^6A	282.1	150.0	6.5	1	140	17	5	Positive
SILIS	C SILIS	253.0	116.0	2.1	1	175	13	5	Positive
	U SILIS	254.0	117.0	2.9	1	95	5	5	Positive
	G SILIS	294.0	157.0	4.1	1	150	17	5	Positive
	A SILIS	278.0	141.0	5.2	1	200	25	5	Positive
	D SILIS	256.0	119.0	1.7	1	70	5	5	Positive
	Ψ SILIS	254.0	218.0	1.7	1	90	5	5	Positive
	m^1A SILIS	295.0	158.0	3.3	2	150	25	5	Positive
	m^5C SILIS	270.0	133.0	3.5	1	185	13	5	Positive
	Cm SILIS	270.0	116.0	3.8	1	180	9	5	Positive
	m^7G SILIS	311.0	174.0	3.8	1	100	13	5	Positive
	I SILIS	279.0	142.0	4.0	1	100	10	5	Positive
	m^5U SILIS	271.0	134.0	4.1	1	95	9	5	Positive
	Gm SILIS	311.0	157.0	4.9	1	100	9	5	Positive
	m^2G SILIS	311.0	174.0	5.0	1	95	17	5	Positive
	m^2_2G SILIS	328.0	191.0	5.6	1	130	13	5	Positive
	m^1G SILIS	311.0	174.0	4.8	1	130	13	5	Positive
	m^6A SILIS	295.0	158.0	6.5	1	140	17	5	Positive

Abbreviations: C, cytidine; U, uridine; G, guanosine; A, adenosine; D, dihydrouridine; Ψ, pseudouridine; m^1A, 1-methyladenosine; m^5C, 5-methylcytidine; Cm, 2'OMe-cytidine; m^7G, 7-methylguanosine; I, inosine; m^5U, 5-methyluridine; Gm, 2'OMe-guanosine; m^2G, 2-methylguanosine; m^2_2G, 2,2-dimethylguanosine; m^1G, 1-methylguanosine; and m^6A, 6-methyladenosine. SILIS: stable isotope labelled internal standard.

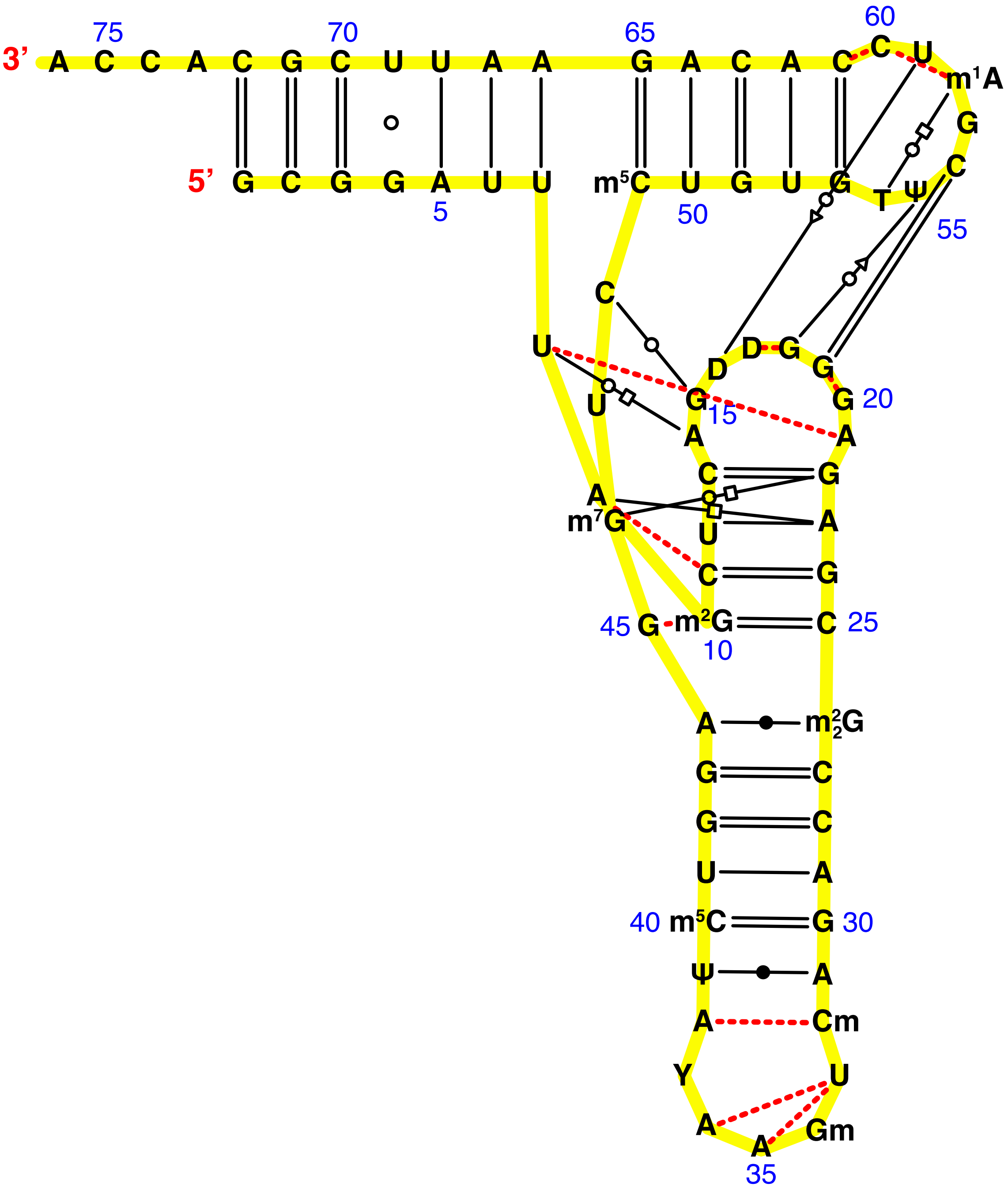

Supplementary Figure 1: Three-dimensional L-shaped structure of yeast tRNA^{Phe}

The L-shaped representation is based on the analysis of the PDB coordinates of yeast tRNA^{Phe} (PDB code 1ehz) (5) with the program RNAMLview (6). The standard Watson-Crick base pairs are annotated with two parallel lines for GC pairs and a single line for AU pairs. GU wobble pairs are annotated with a circle. Single dashed lines mean interactions formed *via* a single hydrogen bond. All the other 12 type of non-Watson-Crick base pairs are annotated with symbols according to the classification defined by Leontis and Westhof (7).

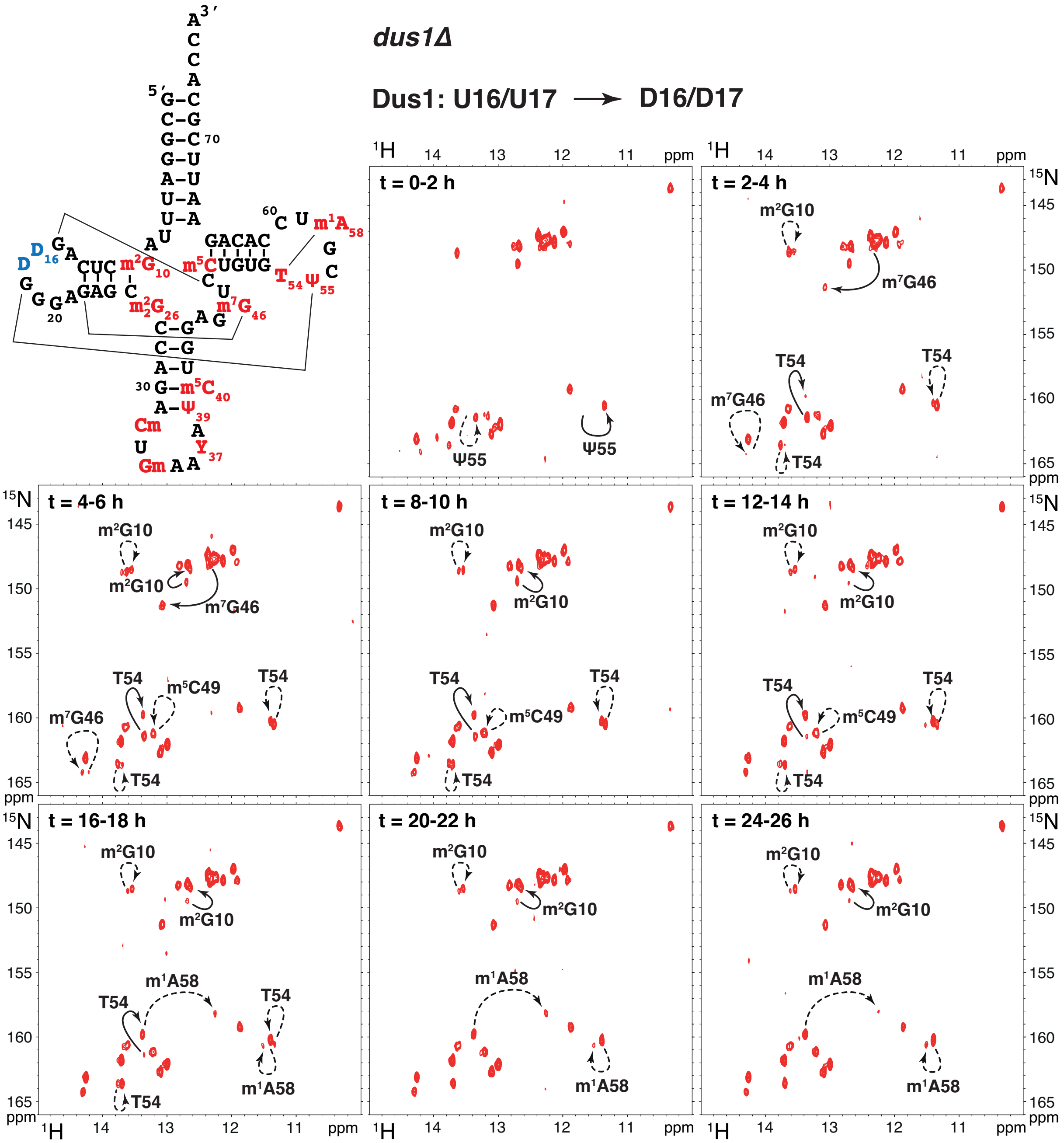

Supplementary Figure 2: Time-resolved NMR monitoring of yeast tRNA^{Phe} maturation in *dus1Δ* yeast extracts

(*top left*) Sequence and cloverleaf representation of modified yeast tRNA^{Phe}. Main tertiary interactions are represented with thin lines. Modifications are in red. The modifications introduced by the Dus1 enzyme and missing in these extracts are in blue.

(*spectra from top to bottom right*) Imino (^{1}H,^{15}N) correlation spectra of a ^{15}N-labelled tRNA^{Phe} measured in a time-resolved fashion during a continuous incubation at 30°C in yeast extract from a *dus1Δ* strain over 26 h. Each NMR spectrum measurement corresponds to a 2 hour time period, as indicated on the top-left corner of each spectrum. Modifications occurring at the different steps are reported with plain arrows for direct effects, or dashed arrows for indirect effects.

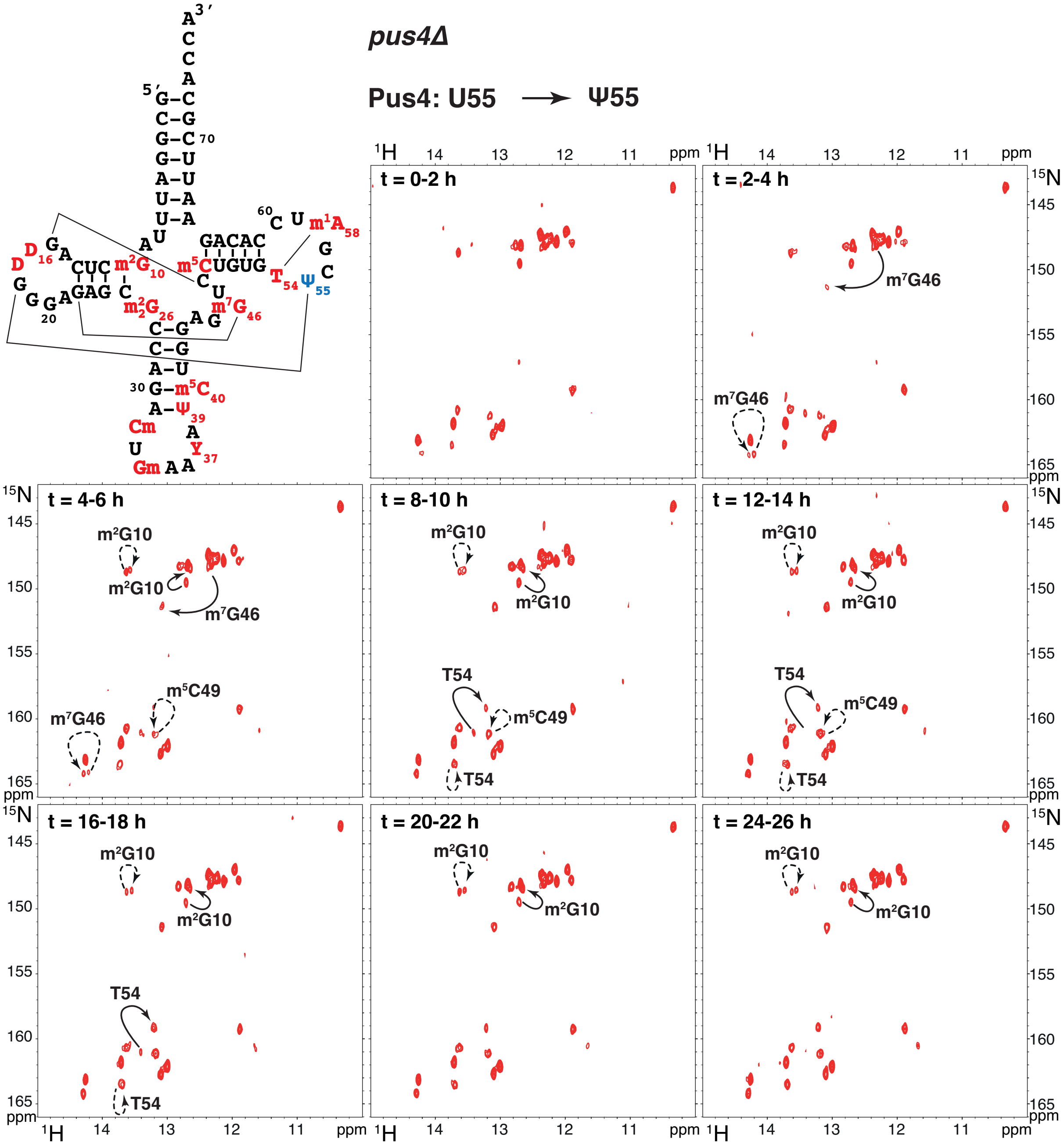

Supplementary Figure 3: Time-resolved NMR monitoring of yeast tRNA^{Phe} maturation in *pus4Δ* yeast extracts

(*top left*) Sequence and cloverleaf representation of modified yeast tRNA^{Phe}. Main tertiary interactions are represented with thin lines. Modifications are in red. The modification introduced by the Pus4 enzyme and missing in these extracts is in blue.

(*spectra from top to bottom right*) Imino (^{1}H,^{15}N) correlation spectra of a ^{15}N-labelled tRNA^{Phe} measured in a time-resolved fashion during a continuous incubation at 30°C in yeast extract from a *pus4Δ* strain over 26 h. Each NMR spectrum measurement corresponds to a 2 hour time period, as indicated on the top-left corner of each spectrum. Modifications occurring at the different steps are reported with plain arrows for direct effects, or dashed arrows for indirect effects.

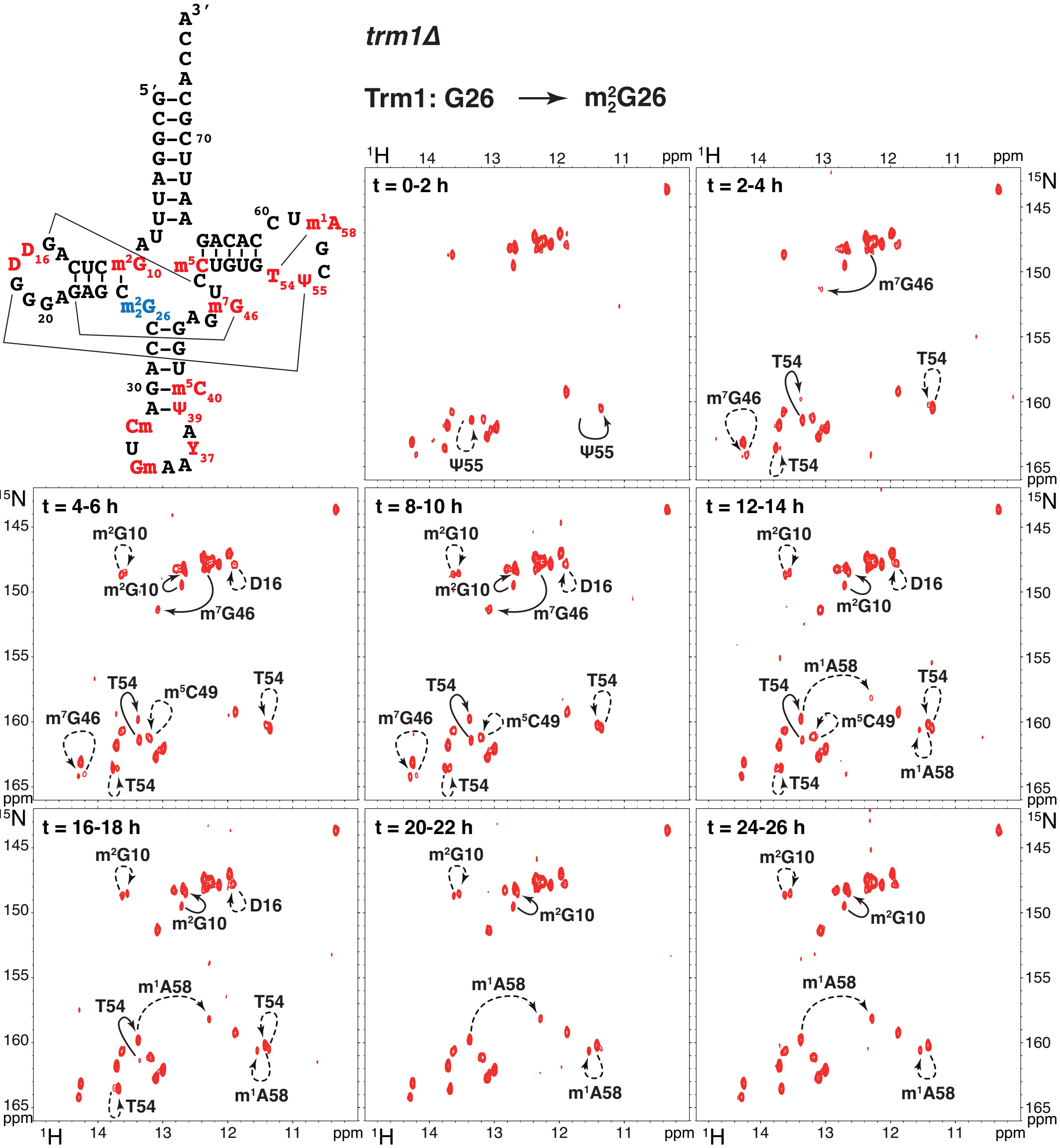

Supplementary Figure 4: Time-resolved NMR monitoring of yeast tRNA^{Phe} maturation in *trm1Δ* yeast extracts

(*top left*) Sequence and cloverleaf representation of modified yeast tRNA^{Phe}. Main tertiary interactions are represented with thin lines. Modifications are in red. The modification introduced by the Trm1 enzyme and missing in these extracts is in blue.

(*spectra from top to bottom right*) Imino (^{1}H,^{15}N) correlation spectra of a ^{15}N-labelled tRNA^{Phe} measured in a time-resolved fashion during a continuous incubation at 30°C in yeast extract from a *trm1Δ* strain over 26 h. Each NMR spectrum measurement corresponds to a 2 hour time period, as indicated on the top-left corner of each spectrum. Modifications occurring at the different steps are reported with plain arrows for direct effects, or dashed arrows for indirect effects.

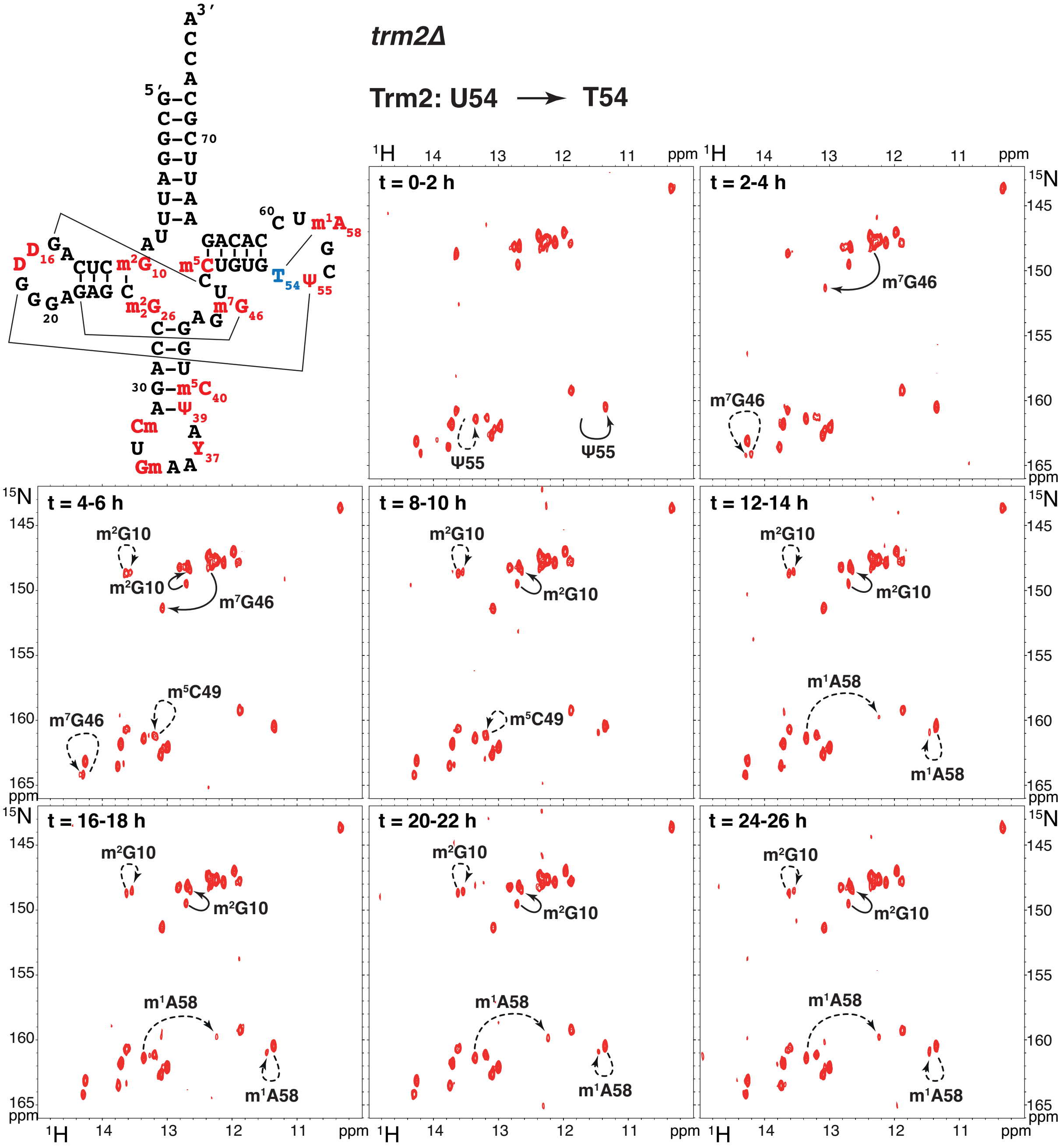

Supplementary Figure 5: Time-resolved NMR monitoring of yeast $tRNA^{Phe}$ maturation in *trm2Δ* yeast extracts

(*top left*) Sequence and cloverleaf representation of modified yeast $tRNA^{Phe}$. Main tertiary interactions are represented with thin lines. Modifications are in red. The modification introduced by the Trm2 enzyme and missing in these extracts is in blue.

(*spectra from top to bottom right*) Imino (^{1}H,^{15}N) correlation spectra of a ^{15}N-labelled $tRNA^{Phe}$ measured in a time-resolved fashion during a continuous incubation at 30°C in yeast extract from a *trm2Δ* strain over 26 h. Each NMR spectrum measurement corresponds to a 2 hour time period, as indicated on the top-left corner of each spectrum. Modifications occurring at the different steps are reported with plain arrows for direct effects, or dashed arrows for indirect effects.

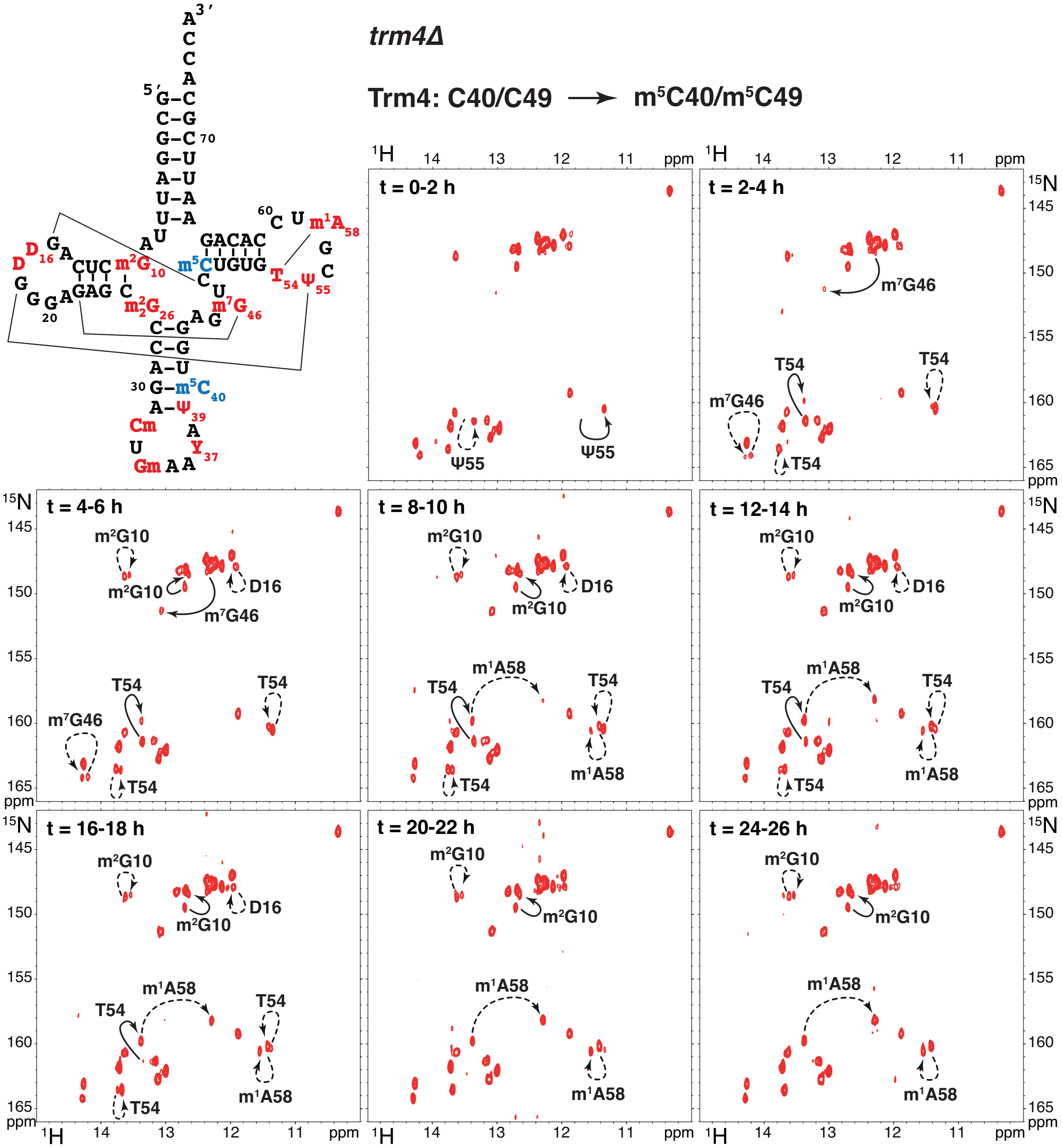

Supplementary Figure 6: Time-resolved NMR monitoring of yeast tRNA^{Phe} maturation in *trm4Δ* yeast extracts

(*top left*) Sequence and cloverleaf representation of modified yeast tRNA^{Phe}. Main tertiary interactions are represented with thin lines. Modifications are in red. The modifications introduced by the Trm4 enzyme and missing in these extracts are in blue.

(*spectra from top to bottom right*) Imino (^{1}H,^{15}N) correlation spectra of a ^{15}N-labelled tRNA^{Phe} measured in a time-resolved fashion during a continuous incubation at 30°C in yeast extract from a *trm4Δ* strain over 26 h. Each NMR spectrum measurement corresponds to a 2 hour time period, as indicated on the top-left corner of each spectrum. Modifications occurring at the different steps are reported with plain arrows for direct effects, or dashed arrows for indirect effects.

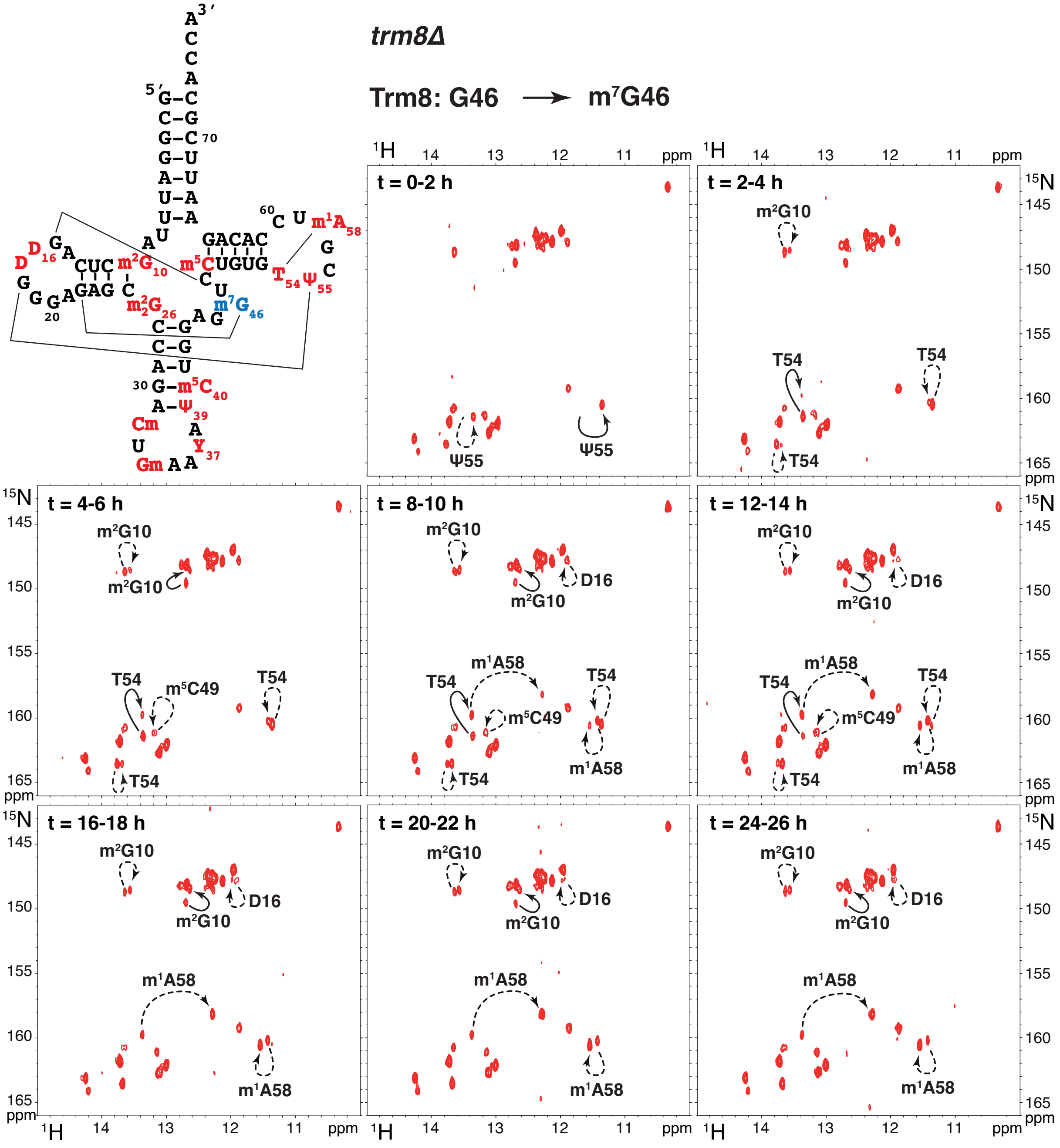

Supplementary Figure 7: Time-resolved NMR monitoring of yeast tRNA^{Phe} maturation in *trm8Δ* yeast extracts

(*top left*) Sequence and cloverleaf representation of modified yeast tRNA^{Phe}. Main tertiary interactions are represented with thin lines. Modifications are in red. The modification introduced by the Trm8 enzyme and missing in these extracts is in blue.

(*spectra from top to bottom right*) Imino (^{1}H,^{15}N) correlation spectra of a ^{15}N-labelled tRNA^{Phe} measured in a time-resolved fashion during a continuous incubation at 30°C in yeast extract from a *trm8Δ* strain over 26 h. Each NMR spectrum measurement corresponds to a 2 hour time period, as indicated on the top-left corner of each spectrum. Modifications occurring at the different steps are reported with plain arrows for direct effects, or dashed arrows for indirect effects.

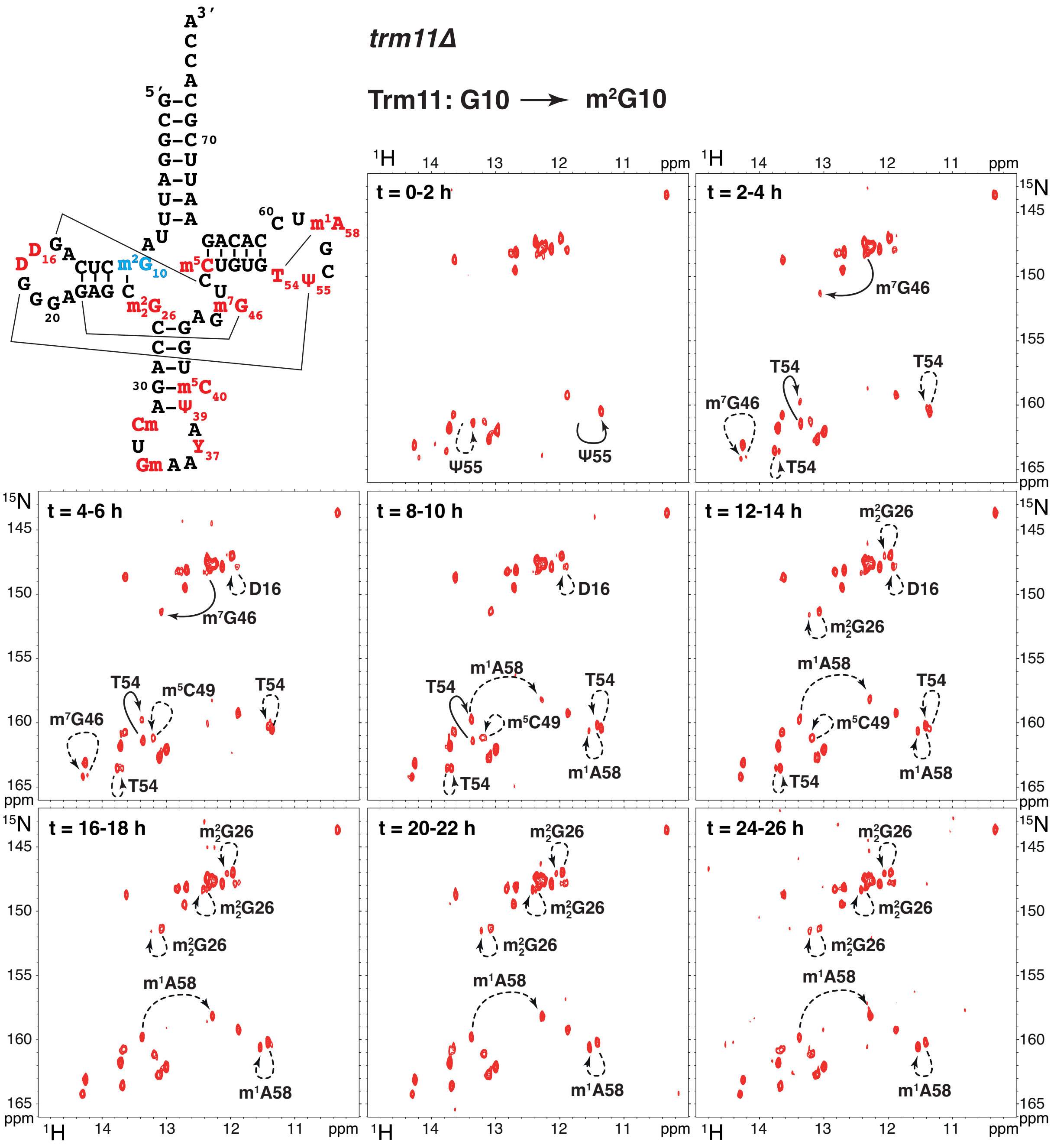

Supplementary Figure 8: Time-resolved NMR monitoring of yeast tRNA^{Phe} maturation in *trm11Δ* yeast extracts

(*top left*) Sequence and cloverleaf representation of modified yeast tRNA^{Phe}. Main tertiary interactions are represented with thin lines. Modifications are in red. The modification introduced by the Trm11 enzyme and missing in these extracts is in blue.

(*spectra from top to bottom right*) Imino (^{1}H,^{15}N) correlation spectra of a ^{15}N-labelled tRNA^{Phe} measured in a time-resolved fashion during a continuous incubation at 30°C in yeast extract from a *trm11Δ* strain over 26 h. Each NMR spectrum measurement corresponds to a 2 hour time period, as indicated on the top-left corner of each spectrum. Modifications occurring at the different steps are reported with plain arrows for direct effects, or dashed arrows for indirect effects.

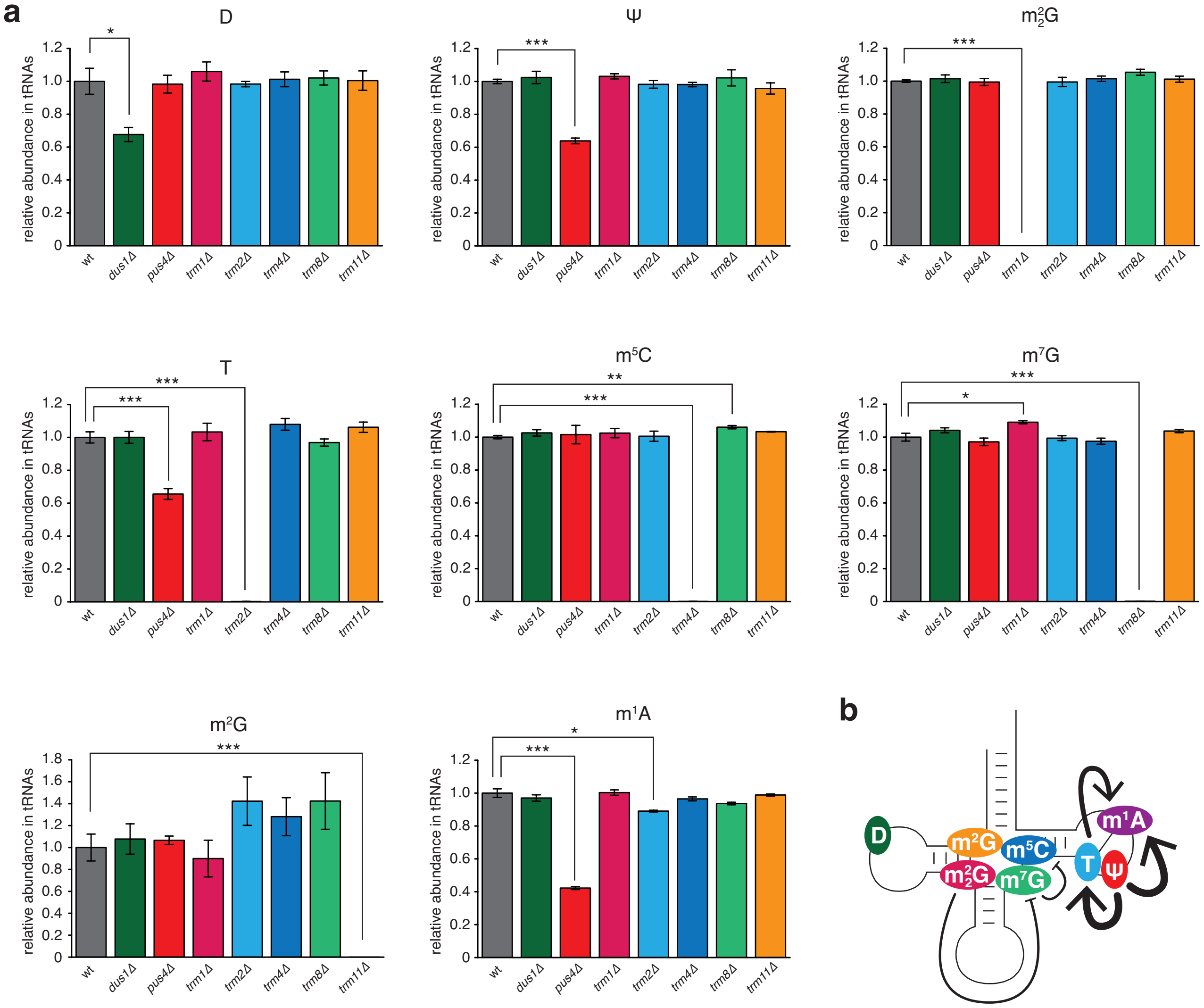

Supplementary Figure 9: Quantitative analysis of nucleoside modifications in yeast total tRNAs with LC-MS/MS

(**a**) Histograms showing the relative abundance of D, Ψ, $m^2{}_2G$, T, m^5C, m^7G, m^2G, and m^1A in total yeast tRNAs prepared from depleted strains (*dus1Δ*, *pus4Δ*, *trm1Δ*, *trm2Δ*, *trm4Δ*, *trm8Δ* and *trm11Δ*) using the wild-type levels as reference. Data heights represent the mean of the biological replicates. Error bars correspond to the s.d. Significant changes compared to wild type are reported as *** for $p<0.001$, ** for $p< 0.01$ and * for $p<0.05$, n=3. All other changes are not statistically significant. Statistical analyses of the variations compared to the wild-type strain were performed using a two-sided Student's t-test. (**b**) Schematic view of the modification circuits revealed by the MS quantification of modifications in yeast total tRNAs. Each modification is displayed on the cloverleaf structure with its associated color. Arrows indicate stimulatory effects and blunted lines inhibitory effects. Thick and thin lines indicate strong and weak effects, respectively. Source data are provided as a Source Data file.

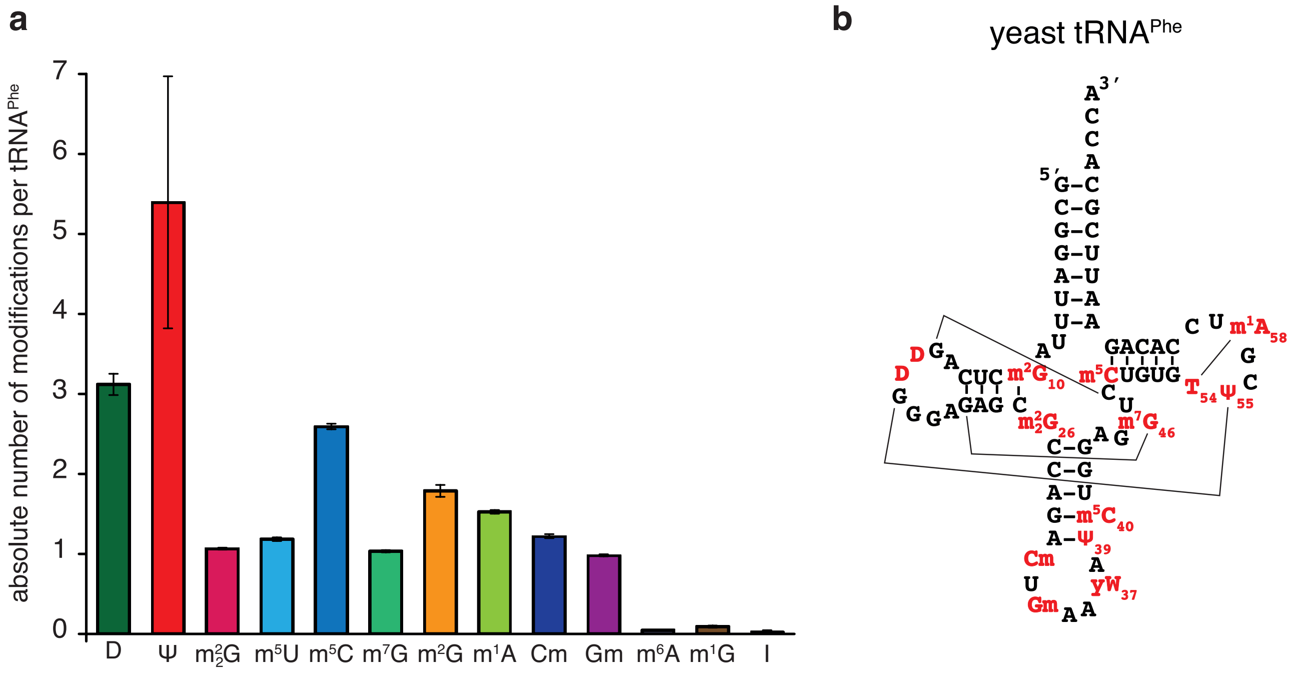

Supplementary Figure 10: Absolute quantification of nucleoside modifications in specifically purified yeast tRNA^{Phe} with LC-MS/MS

(**a**) Histograms showing the absolute abundance of modified nucleosides in tRNA^{Phe} isolated from wild-type yeast. Data heights represent the mean of the biological replicates (n=3). Error bars correspond to the s.d. Due to chromatographic separation issues and low mass spectrometric detection efficiency, the quantification of pseudouridine is disturbed, which leads to an overestimation of pseudouridine in the measurements (also reflected in large error bars) (**b**) Sequence and cloverleaf representation of yeast tRNA^{Phe}. Modifications are in red. Source data are provided as a Source Data file.

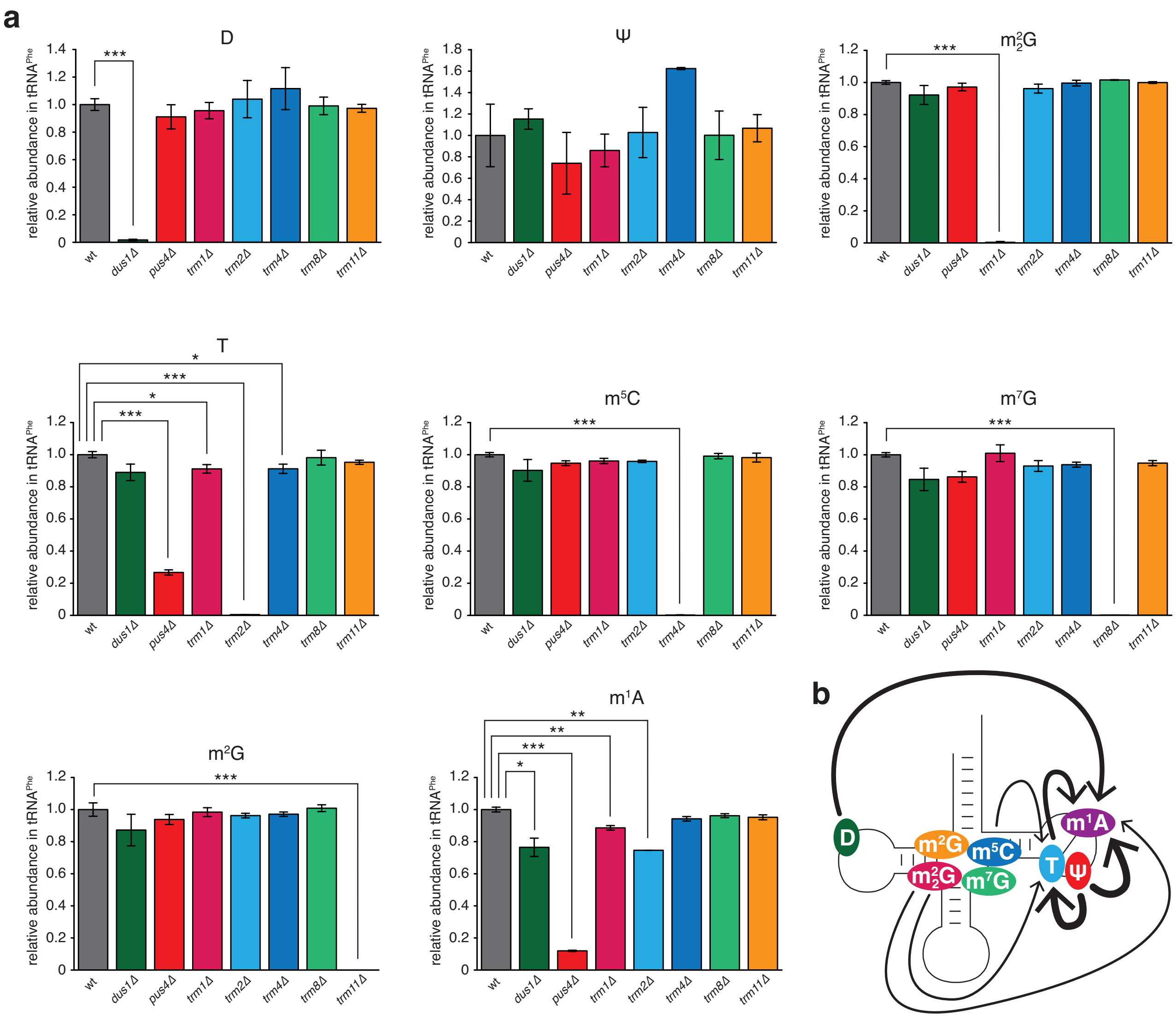

Supplementary Figure 11: Quantitative analysis of nucleoside modifications in specifically purified yeast tRNA^{Phe} with LC-MS/MS

(**a**) Histograms showing the relative abundance of D, Ψ, $m^2{}_2G$, T, m^5C, m^7G, m^2G, and m^1A in specifically purified yeast tRNA^{Phe} prepared from depleted strains (*dus1Δ*, *pus4Δ*, *trm1Δ*, *trm2Δ*, *trm4Δ*, *trm8Δ* and *trm11Δ*) using the wild-type levels as reference. Data heights represent the mean of the biological replicates. Error bars correspond to the s.d. Significant changes compared to wild type are reported as *** for p<0.001, ** for p< 0.01 and * for p<0.05, n=3. All other changes are not statistically significant. Statistical analyses of the variations compared to the wild-type strain were performed using a two-sided Student's t-test. Modifications were quantified in 3 independent biological replicates, except for the modifications Ψ, m^7G and m^1A of the *trm4Δ* strain, for which n=2. (**b**) Schematic view of the modification circuits revealed by the MS quantification of modifications in specifically purified yeast tRNA^{Phe}. Each modification is displayed on the cloverleaf structure with its associated color. Arrows indicate stimulatory effects. Thick and thin lines indicate strong and weak effects, respectively. Source data are provided as a Source Data file.

Supplementary References

1. Ohira, T. & Suzuki, T. Retrograde nuclear import of tRNA precursors is required for modified base biogenesis in yeast. *Proc Natl Acad Sci U S A* **108,** 10502-7 (2011).
2. Hopper, A. K., Furukawa, A. H., Pham, H. D., & Martin, N. C. Defects in modification of cytoplasmic and mitochondrial transfer RNAs are caused by single nuclear mutations. *Cell* **28,** 543-50 (1982).
3. Nordlund, M. E., Johansson, J. O., von Pawel-Rammingen, U., & Byström, A. S. Identification of the TRM2 gene encoding the tRNA(m5U54)methyltransferase of Saccharomyces cerevisiae. *RNA* **6,** 844-60 (2000).
4. Heinemeyer, W., Kleinschmidt, J. A., Saidowsky, J., Escher, C., & Wolf, D. H. Proteinase yscE, the yeast proteasome/multicatalytic-multifunctional proteinase: mutants unravel its function in stress induced proteolysis and uncover its necessity for cell survival. *EMBO J* **10,** 555-62 (1991).
5. Shi, H. & Moore, P. B. The crystal structure of yeast phenylalanine tRNA at 1.93 A resolution: a classic structure revisited. *RNA* **6,** 1091-105 (2000).
6. Yang, H., Jossinet, F., Leontis, N., Chen, L., Westbrook, J., Berman, H., & Westhof, E. Tools for the automatic identification and classification of RNA base pairs. *Nucleic Acids Res* **31,** 3450-60 (2003).
7. Leontis, N. B. & Westhof, E. Geometric nomenclature and classification of RNA base pairs. *RNA* **7,** 499-512 (2001).
